# Supplementary material for: AffyMAPSDetector: a software tool to characterize Affymetrix GeneChip™ expression arrays with respect to SNPs
Source: BMC Bioinformatics. 2007 Jul 30;8:276. doi: 10.1186/1471-2105-8-276 (PMC1959249; doi:10.1186/1471-2105-8-276)
Supplement: Additional file 13 — AffyMAPSDetector v1 source code. AffyMAPSDetector source code. [file 1471-2105-8-276-S13.zip › AffyMAPSDetector_v1_src/doc/affychip/snpdetector/model/SnpDetector.html]

SnpDetector


|  |  |  |  |
| --- | --- | --- | --- |
| **Overview** \| **Package** \| **Class** \| **Use** \| **Tree** \| **Deprecated** \| **Index** \| **Help** | | |  |
| **PREV CLASS**   **NEXT CLASS** | **FRAMES**    **NO FRAMES**     **All Classes** |  |  |
| SUMMARY: NESTED \| FIELD \| CONSTR \| METHOD | DETAIL: FIELD \| CONSTR \| METHOD |  |  |


---


## affychip.snpdetector.model Class SnpDetector

```
java.lang.Object
  affychip.snpdetector.model.SnpDetector
```

**Direct Known Subclasses:**: OptimizedSnpDetector

---

public class **SnpDetector** extends java.lang.Object

Title: Probe SNP Detector

Test program that accesses NCBI SNP database online and looks for SNPs in
a given gene whose locus-link-id and gene-id is known.

Copyright: Copyright (c) 2004

Company: George Mason University

**Version:**
:   1.1

**Author:**
:   Sunita Kumari

---

|  |  |
| --- | --- |
| **Field Summary** | |
| `static java.lang.String` | `findLocusLinkForAccessionNo` |
| `static java.lang.String` | `ncbiGeneAccessionNoSearchUrl` |
| `static java.lang.String` | `ncbiNucleotideDbUrl` |
| `static java.lang.String` | `ncbiRefSnpClusterUrl` |
| `static java.lang.String` | `ncbiSnpDbUrl` |


**Constructor Summary**

`SnpDetector()`


|  |  |
| --- | --- |
| **Method Summary** | |
| `GeneInfo` | `dectectSnpsFor(java.lang.String geneAccessionNumber, java.lang.String locusLinkId)`             THIS METHOD IS USED ONLY FOR EXPERIMENT AND TESTING. |
| `void` | `detectSnpsFor(GeneInfo geneInfo)`             This method accesses a gene with a given accession number (from GenBank) and locus-link-id online and searches NCBI snp database for existence of SNPs in this gene's nucleotide-sequence. |
| `int` | `findLocusLinkForAccessionNumber(java.lang.String accNo)` |
| `static void` | `main(java.lang.String[] args)` |

**Methods inherited from class java.lang.Object**

`clone, equals, finalize, getClass, hashCode, notify, notifyAll, toString, wait, wait, wait`

**Field Detail**

### ncbiSnpDbUrl

```
public static final java.lang.String ncbiSnpDbUrl
```

**See Also:**: Constant Field Values

---


### ncbiNucleotideDbUrl

```
public static final java.lang.String ncbiNucleotideDbUrl
```

**See Also:**: Constant Field Values

---


### ncbiRefSnpClusterUrl

```
public static final java.lang.String ncbiRefSnpClusterUrl
```

**See Also:**: Constant Field Values

---


### ncbiGeneAccessionNoSearchUrl

```
public static final java.lang.String ncbiGeneAccessionNoSearchUrl
```

**See Also:**: Constant Field Values

---


### findLocusLinkForAccessionNo

```
public static final java.lang.String findLocusLinkForAccessionNo
```

**See Also:**: Constant Field Values


**Constructor Detail**

### SnpDetector

```
public SnpDetector()
```


**Method Detail**

### dectectSnpsFor

```
public GeneInfo dectectSnpsFor(java.lang.String geneAccessionNumber,
                               java.lang.String locusLinkId)
```

:   THIS METHOD IS USED ONLY FOR EXPERIMENT AND TESTING. WRITE YOUR
    EXPERIMENTAL CODE HERE AND TEST THAT BY CALLING THIS METHOD FROM main().
    This method accesses a gene with a given accession number (from GenBank)
    and locus-link-id online and searches NCBI snp database for existence of
    SNPs in this gene's nucleotide-sequence.

    :   **Parameters:**: `geneAccessionNumber` - GenBank Accession Number of the gene to look SNPs for.: `locusLinkId` - Locus Link Id of the gene to look SNPs for. **Returns:**: semi-colon separated list of SNPs locations if SNP(s) exist, null otherwise.

---


### findLocusLinkForAccessionNumber

```
public int findLocusLinkForAccessionNumber(java.lang.String accNo)
```

---


### detectSnpsFor

```
public void detectSnpsFor(GeneInfo geneInfo)
```

:   This method accesses a gene with a given accession number (from GenBank)
    and locus-link-id online and searches NCBI snp database for existence of
    SNPs in this gene's nucleotide-sequence. Both parameters are retrieved
    from the passed GeneInfo.

    :   **Parameters:**: `geneInfo` - type GeneInfo

---


### main

```
public static void main(java.lang.String[] args)
```


---


|  |  |  |  |
| --- | --- | --- | --- |
| **Overview** \| **Package** \| **Class** \| **Use** \| **Tree** \| **Deprecated** \| **Index** \| **Help** | | |  |
| **PREV CLASS**   **NEXT CLASS** | **FRAMES**    **NO FRAMES**     **All Classes** |  |  |
| SUMMARY: NESTED \| FIELD \| CONSTR \| METHOD | DETAIL: FIELD \| CONSTR \| METHOD |  |  |


---
